# *Schistosoma japonicum* Infection Promotes the Response of Tfh Cells Through Down-Regulation of Caspase-3–Mediating Apoptosis

**DOI:** 10.3389/fimmu.2019.02154

**Published:** 2019-09-13

**Authors:** Quan Yang, Jiale Qu, Chenxi Jin, Yuanfa Feng, Shihao Xie, Jinxin Zhu, Gaoshen Liu, Hongyan Xie, Huaina Qiu, Yanwei Qi, Jianbing Mu, Jun Huang

**Affiliations:** ^1^Guangdong Provincial Key Laboratory of Allergy and Clinical Immunology, The State Key Laboratory of Respiratory Disease, The Second Affiliated Hospital, Guangzhou Medical University, Guangzhou, China; ^2^Laboratory of Malaria and Vector Research, National Institute of Allergy and Infectious Diseases, National Institutes of Health, Bethesda, MD, United States; ^3^Sino-French Hoffmann Institute, Guangzhou Medical University, Guangzhou, China

**Keywords:** *Schistosoma japonicum*, T follicular helper (Tfh) cells, apoptosis, caspase-3, SEA

## Abstract

CD4^+^ T follicular helper (Tfh) cells, a new subset of immune cells, have been demonstrated to be involved in granulomatous responses to *Schistosoma japonicum (S. japonicum)* infection. However, the role and underlying mechanisms of Tfh cell aggregation in *S. japonicum* infection remain incompletely understood. In this study, we provide evidence that *S. japonicum* infection enhances the accumulation of Tfh cells in the spleen, lymph nodes, and peripheral blood of C57BL/6 mice. Infection-induced Tfh cells exhibited more potent effects directly on B cell responses than the control Tfh cells (*P* < 0.05). Furthermore, reduced apoptosis of Tfh cells was found both in *S. japonicum* infected mice and in soluble egg antigen (SEA) treated Tfh cells (*P* < 0.05). Mechanistic studies reveal that caspase-3 is the primary drivers of down-regulated apoptotic Tfh cell death in *S. japonicum* infection. In summary, this study demonstrates that Tfh cell accumulation might have an impact on the generation of immune responses in *S. japonicum* infection, and caspase-3 signaling mediated apoptosis down-regulation might responsible for the accumulation of Tfh cell in this course.

## Introduction

Schistosomiasis remains a devastating public health problem that affects about 240 million people worldwide and more than 700 million persons are at risk of infection, especially in developing countries (1). Once infected, the eggs released by *S. japonicum* will deposited in the body, such as liver, lung, and enteric wall, which later cause the organ pathologies including granuloma and fibrosis, and finally lead to organ failure (2). The deposited worm eggs secreting soluble egg antigen (SEA) could induce a Th2 dominant humoral immune response in both infected human and animals host (3). Many different types of immune cells, effector molecules, and numerous cytokines are involved in the development or progression of the disease (4–7).

Follicular helper T (Tfh) cells are specialized providers of T cell help to B cells, and are essential for germinal center formation, affinity maturation, and the development of most high-affinity antibodies and memory B cells (8). Although, there is no single marker for distinguishing Tfh cells from other CD4 subsets, they are defined by their expression of surface co-stimulatory molecules CXCR5, CD200, ICOS, and a high density of PD-1 (9). It has been shown that ICOS in Tfh cells can promote the activation of B cells by interacting with ICOSL, which is highly expressed on the surface of B cells, and up-regulate the expression of BcL-6, a key transcription factor for Tfh cell differentiation (10). In addition, IL-21 is the most important molecule to facilitate Tfh cells to assist in the differentiation and function of B cells. Kong et al. found that increased CD4^+^CXCR5^+^ Tfh cells could promote the enrichment of CD27^+^IgG^+^ B cells through IL-21 secretion (11).

Recently, Tfh cells have emerged to play a beneficial role in mediating anti-parasitic immunity including the resistance to schistosome infections (12). For example, Chen et al. found that after *S. japonicum* infection, Tfh cells were recruited into the liver in large quantities to promote the formation of granuloma (13). It is well-known that when the host is infected by the parasite, the number of immune cells can increase dramatically due to either the expansion of cell division (extension of cell life) (14) or the decrease in cell apoptosis (15). Apoptosis can occur in the whole process of lymphocyte development and differentiation (16), and is regulated by the relevant signal transduction pathway. Caspase-3, a key enzyme and executor of apoptosis (17), belongs to the cysteine protease family and is an important downstream effector protease of T-cell apoptosis. It is widely believed that the mRNA level of caspase-3 is an important indicator of cell apoptosis (18). When a cell is affected by an immunosuppressant, caspase-3 is considered to be the most suitable indicator for apoptosis assessment of any T-cell subgroup (19). In most cases, caspase-3 exists in the form of procaspase-3, which is activated to initiate apoptosis (17). It is believed that procaspase activating compound-1 (PAC-1) protein is one of the strongest activators of procaspase-3, which can promote the self-activation of procaspase-3 and induce apoptosis by chelating zinc ions (20). Caspase-3 can regulate the activity of many kinds of anti-apoptotic associated genes, such as *Mcl-1, XIAP*, and *Bcl-2* (21). Apoptotic *Bcl-2* members such as *Bcl-2, Bcl-xL*, and *Mcl-1* can lead to defective apoptosis, resulting in enhancing cell survival and drug resistance (22). BAX is an intrinsic apoptosis effector that is wildly used, especially in chemical drug research (23, 24). *XIAP* can impair mitochondrial function during apoptosis by regulating the *Bcl-2* family in renal cell carcinoma (25). Down-regulation of *survivin* expression can induce apoptosis in renal cancer Caki cells (26).

In human schistosome infection, an induce apoptosis of CD4^+^ Th cells was reported previously (27). In addition, Wang et al. found that SEA could induce apoptosis of hepatic stellate cells by down-regulating Akt expression and up-regulating DR5 expression dependent on p53, in combating liver fibrosis caused by *S. japonicum* infection (28). Whether, the apoptosis plays a significant role in Tfh accumulation during *S. japonicum* infection remains elusive. In this study, we, therefore, systematically investigated the role of Tfh cells in the course of *S. japonicum* infected C57BL/c mice, and explored the possible mechanism of Tfh cells accumulation by focusing on the Tfh apoptosis.

## Materials and Methods

### Mice, Parasites, and Infection

Female C57BL/6 mice were purchased from the Animal Experimental Center of Sun Yat-Sen University (Guangzhou, China). All mice were maintained under specific pathogen-free conditions and used at 6–8 weeks of age. *S. japonicum* cercariae were shed from naturally infected *Oncomelania hupensis* snails, which were purchased from Jiangsu Institute of Parasitic Disease (Wuxi, China). C57BL/6 mice were infected percutaneously with 40 ± 5 cercariae, and they were sacrificed at 5–6 weeks after infection. Animal experiments were performed in strict accordance with the regulations for the Administration of Affairs Concerning Experimental Animals, and all efforts were made to minimize suffering.

### Reagents and Antibodies

RPMI 1640, FBS, penicillin, and streptomycin were obtained from Invitrogen (Grand Island, NY). Recombinant murine IL-21 was purchased from Peprotech (Oak Park, CA). The antibodies against caspase-3, caspase-8, β-actin, and HRP-conjugated secondary antibodies were purchased from Santa Cruz Biotechnology (Santa Cruz, CA). Phorbol 12-myristate 13-acetate (PMA), Brefeldin A, ionomycin, CD3, CD28, and dimethyl sulfoxide (DMSO) were purchased from Sigma-Aldrich (St. Louis, MO). We obtained the following fluorescein-conjugated anti-mouse antibodies from eBioscience (San Diego, CA) or biolegend (San Diego, CA): CD3e-PE-Cy7 (145-2C11), CD4-PerCP-5.5 (GK1.5), PD-1-APC (J43), PD-1-PE-Cy7 (eBioJ105 (J105)), CXCR5-BV421 (J252D4), ICOS-PE-Cy7 (C398.4A), CD25-PE (PC61.5), CD69-FITC (H1.2F3), CD19-APC-Cy7(6D5), CD27-FITC (B30C7), IL21-PE (MHALX21), IL10-APC (JES5-16E3), IFN-γ-APC (XMG1.2), IL17-PE (eBio17B7), IL4-PE (11B11), and IL5-APC (TRFK5) and their corresponding isotype controls. The Annexin V-FITC/PI Apoptosis Detection Kit and anti-caspase-3-FITC (C92-605) were obtained from BD Biosciences (San Jose, CA). The caspase-3 activator PAC-1 (315183-21-2) was purchased from Selleck (Houston, TX).

### SEA Preparation

SEA of *S. japonicum* cercariae were obtained from Jiangsu Institute of Parasitic Diseases (China) (29). SEA was sterile-filtered, and endotoxin was removed with the use of Polymyxin B agarose beads (Sigma). The Limulus amoebocyte lysate assay kit (Lonza, Switzerland) was used to confirm the removal of endotoxins from the SEA.

### Histology Studies

Parts of livers were cut and perfused three times with 0.01 M phosphate-buffered saline (pH = 7.4), fixed in 10% formalin, embedded in paraffin, and sectioned. The slice was stained by standard hematoxylin-eosin (H&E) staining, and examined by light microscopy under 100× magnification.

### Lymphocyte Isolation

Mice were sacrificed, peripheral blood (PB) was collected, liver, spleen (SP), and mesenteric lymph nodes (LN) were removed. Spleen, and MLN were mechanically dissociated and processed through a 100-um cell strainer (BD Falcon), and suspended in HBSS. Lymphocytes were isolated by Ficoll-Hypaque (DAKEWE, SZ, China) density gradient centrifugation from cell solution and diluted in blood solution. Isolated cells were washed twice in HBSS and re-suspended at 2 × 10^6^ cells/ml in complete RPMI 1640 medium supplemented with 10% heat-inactivated fetal calf serum (FCS), 100 U/ml penicillin, 100 μg/ml streptomycin, 2 mM glutamine, and 50 μM 2-mercaptoethanol.

### Quantitative Real-Time PCR (qRT-PCR) and Western Blotting

These experiments were performed following previously described procedures (7). Briefly, RNA was extracted with an RNase Mini Kit and cDNA was synthesized with a SuperScript III Reverse Transcriptase (QIAGEN, Valencia, CA, USA). qRT-PCR was performed with 2.5 μL of cDNA, 12.5 μL of SYBR Master Mixture (Applied Biosystems, Foster City, CA, USA), and target gene–specific primers (Table 1). Amplification of β-actin was used as an internal control. For western blotting, the cultured or purified cells were collected and lysed. The protein concentration was measured with a bicinchoninic acid protein assay kit (Beyotime). The protein sample was separated in 10% SDS-denatured polyacrylamide gel and transferred onto a polyvinylidene difluoride membrane. The polyvinylidene difluoride membranes were blocked with 5% skim milk in TBST at room temperature for 2 h. The targeted molecules were probed using specific primary antibodies and HRP-conjugated secondary antibodies and detected with an ECL HRP chemiluminescent substrate reagent kit (Invitrogen, Carlsbad, CA).

**Table 1:** Sequences of primers.

**Gene**	**Forward Primer (5'-3')**	**Reversed Primer (5'-3')**
BAX	TGGAGATGAACTGGACAGCA	GAAGTTGCCATCAGCAAACA
Survivin	GGCAGCTGTACCTCAAGAA	TCTATCGGGTTGTCATCGGG
XIAP	ACCCTGCCATGTGTAGTGAA	ACGATCACAGGGTTCCCAAT
Bcl-2	CTTCAGGGATGGGGTGAACT	TACTCAGTCATCCACAGGGC
Mcl-1	GATGGCGTAACAAACTGGGG	AACTCCACAAACCCATCCCA
GATA3	GGCCAGGCAAGATGAGAAAG	AGGGCGGATAGGTGGTAATG
Bcl-6	GACGCACAGTGACAAACCAT	AACTGCGCTCCACAAATGTT
BATF	AAGAGCCGACAGAGACAGA	TCCTCGGTGAGCTGTTTGAT
T-bet	GTGTCTGGGAAGCTGAGAGT	GGTGAAGGACAGGAATGGGA
MAF	GCGCACCTGGAAGACTACTA	GCATAGCCATCGGAAGCCAC
β-actin	CCGTAAAGACCTCTATGCCAAC	GGGTGTAAAACGCAGCTCAGTA

### Cell Surface Staining and Cell Population Isolation

Cells were washed twice in PBS and blocked in PBS buffer containing 1% BSA for 30 min. Then, the cells were stained with conjugated antibodies that were specific for cell surface antigens for 30 min at 4°C in the dark. These antigens included CD3e, CD4, PD-1, CXCR5, ICOS, CD25, CD69, CD19, IgD, and CD27. The stained lymphocytes were analyzed by using flow cytometry (Beckman Coulter, Fullerton, CA), and the results were analyzed with use of the software CytoExpert 2.0 (Beckman Coulter). Isotype-matched cytokine controls were included in each staining protocol. For purification of the Tfh cells, mouse splenocytes were stained with CD4-PerCP-5.5, CXCR5-FITC, and PD-1-BV421 antibodies, and CD4^+^CXCR5^+^PD-1^+^ cells were isolated by cell sorting on a FACS Aria cell sorter (BD, Mountain View, CA). For purification of the-B cells, mouse splenocytes were stained with CD19-PE-cy7, and CD19^+^ B cells were isolated by cell sorting on a FACS Aria cell sorter (BD, Mountain View, CA).

### Cell Intracellular Cytokine and Molecule Staining

Single-cell suspensions from the spleens of control mice and mice infected with *S. japonicum* were stimulated with 20 ng/mL phorbol 12-myristate 13-acetate (PMA) plus 1 μg/mL ionomycin for 5 h at 37°C under a 5% CO_2_ atmosphere. Brefeldin A (10 g/mL, Sigma, Shanghai, China) was added during the last 4 h of incubation. Cells were washed twice in PBS, fixed with 4% paraformaldehyde, and permeabilized overnight at 4°C in PBS buffer containing 0.1% saponin (Sigma), 0.1% BSA, and 0.05% NaN_3_. Cells were then stained for 30 min at 4°C in the dark with conjugated antibodies specific for the cell surface antigens CD4, CXCR5, and PD-1 as well as the intracellular cytokines or proteins IL-21, IL-10, IFN-γ, IL-17, IL-4, and IL-5. For staining of BcL-6 and caspase-3: cells were isolated and fixed and permeabilized overnight at 4°C by Fixation/Permeabilization Solution according to the manufacturer's instructions (BD, San Jose, CA). Different fluorescence labeled antibody was added, and stained for 30 min at 4°C in the dark. The expression phenotypes of the antibody-labeled lymphocytes were analyzed by flow cytometry (Beckman Coulter, Fullerton, CA), and the results were analyzed with the software CytoExpert 2.0 (Beckman Coulter). Isotype-matched cytokine controls were included in each staining protocol.

### Tfh Cell and B Cell Co-culture

Naive B cells were co-cultured with Tfh cells (2 × 10^5^ cells per well) at a ratio of 1:1 in RPMI 1640 complete medium supplemented with 10% fetal bovine serum. Culture medium was collected at day 3 or 10, and the levels of immunoglobulin G (IgG) and IgM were determined by enzyme-linked immunosorbent assay (ELISA) according to the manufacturer's instructions (Bethyl Laboratories). The expression of CD27, and CD69 on B cells was detected using cell surface staining.

### Annexin V-FITC/Propidium Iodide Staining

For apoptosis assays, C57BL/6 mice were infected percutaneously with or without 40 ± 5 cercariae as described earlier. At 5–6 weeks after infection, the mice were sacrificed, and the spleens were collected for analysis of apoptotic cells. Cells were initially stained using the makers for Tfh cells (CD4^+^PD-1^+^CXCR5^+^), washed, and then stained with the annexin V-FITC and propidium iodide (PI) according to the manufacturer's instructions (BD Pharmingen).

### Measurement of Caspase-3 Activity

The activity of caspase-3 was measured using the caspase-3 Activity Assay Kit (Beyotime, China). Following the manufacturer's instructions, a standard curve was generated by measuring the absorbance (A_405_) of varying amounts of standard p-nitroaniline (pNA). Then, the Tfh cells were purified from normal or *S. japonicum* infected mice, and the proteins were harvested. Acetyl-Asp-Glu-Val-Asp *p*-nitroanilide (Ac-DEVD-pNA) was then added to each sample at 37°C for 2 h. The absorbance was determined on an ELISA reader (BioTek, USA) and converted to the amounts of pNA that were produced in the cells.

### Statistics

Statistical analysis between different groups was performed using unpaired *t*-tests. One-way ANOVA was used to analysis the dynamic proportions of splenic Tfh cells. The software packages GraphPad Prism version 5.0a and SPSS Statistics 17.0 were used. *P* < 0.05 was considered statistically significant.

## Results

### Accumulation of Tfh Cells in *Schistosoma japonicum* Infected Mice

**Figure 1:**
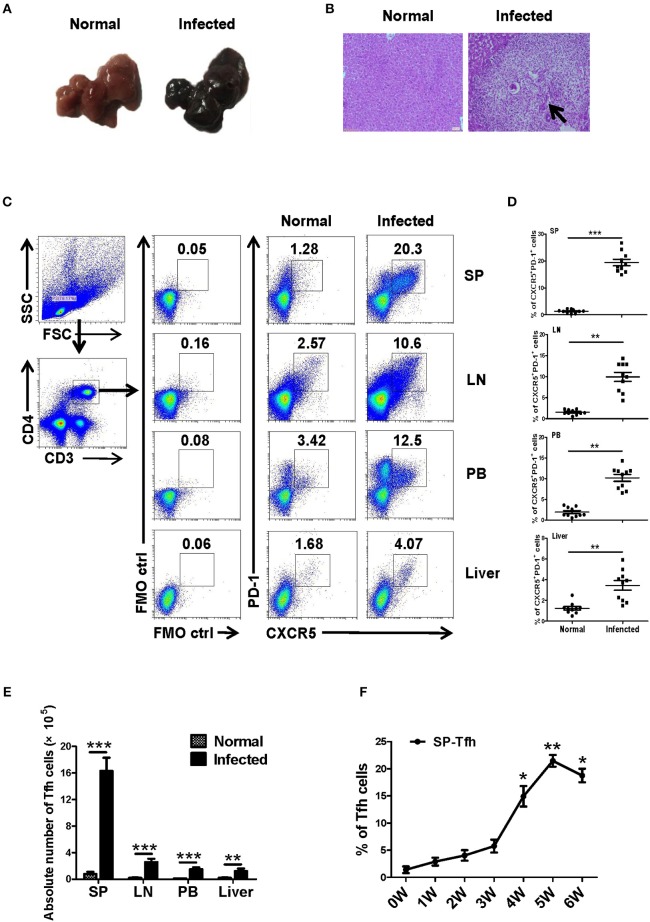
*S. japonicum*-infection induce Tfh cells *in vivo*. **(A–D)** C57BL/6 mice were infected percutaneously with 40 ± 5 cercariae and sacrificed at 5–6 weeks after infection, and different tissues were harvested. **(A)** Representative images of livers. **(B)** Representative images of liver H&E staining; arrows indicate granuloma. The percentage **(C,D)** and absolute numbers **(E)** of CD4^+^CXCR5^+^PD1^+^ cells in the lymphocytes isolated from spleen (SP), lymph node (LN), peripheral blood (PB), and live of both normal and infected mice were evaluated by flow cytometry after staining with specific antibodies. Each group included 10 mice. **(F)** Spleens from infected mice were harvested; the dynamic proportions of CD4^+^CXCR5^+^PD-1^+^ Tfh cells in the *S. japonicum* infected mice was evaluated by flow cytometry. Data are shown as mean ± SEM of six samples in each group from one representative experiment, and repeat three times with similar results. ^*^P < 0.05, ^**^P < 0.01, ^***^P < 0.001 compared with the corresponding controls (0W), One-way ANOVA was used.

Twenty female SPF C57BL/6 mice (6–8 weeks old) were randomly divided into the infection group and the control group, with 10 mice in each group. The infected mice were infected with *S. japonicum* (40 ± 5) through the abdominal skin. At 5–6 weeks later, liver lesions and granuloma were observed in infected mice (Figures 1A,B). Lymphocytes from different immune organs, such as liver, spleen and lymph node, and peripheral blood were isolated from mice in the control and the infected groups and stained as described in Materials and Methods. The proportions of Tfh (CD4^+^CXCR5^+^PD-1^+^ cells) in each group were detected by flow cytometry. Results showed that the ratio and absolute number of Tfh cells in the organs of the infected mice increased significantly (*P* < 0.05, Figures 1C–E). Furthermore, the dynamic proportions of splenic CD4^+^CXCR5^+^PD-1^+^ Tfh cells in the *S. japonicum* infected mice was evaluated by flow cytometry. The result showed that dynamic up–regulation of splenic Th cells was observed during the course of *S. japonicum* infection (Figure 1F).

### Activation of Tfh Cells From *S. japonicum* Infected Mice

**Figure 2:**
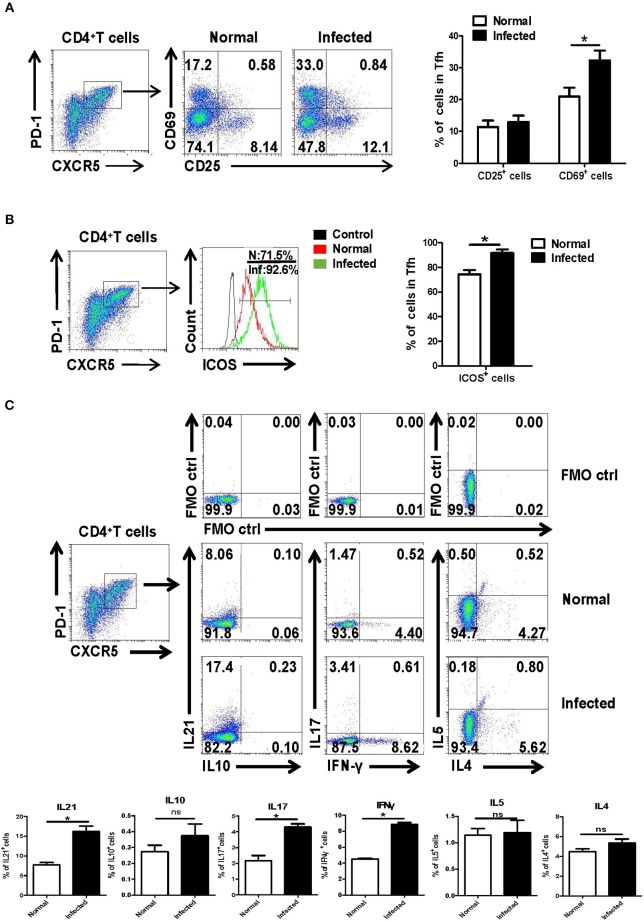
Activation associated molecules and cytokines expressing on Tfh cells. Single-cell suspensions of spleens were isolated from normal and infected mice. The expression of CD25 and CD69 on Tfh cells was detected using cell surface staining. The expression of CD25, CD69 **(A)**, and ICOS **(B)** was analyzed using FCM; average expression of CD25, CD69, and ICOS was calculated from FACS data. Three independent experiments with similar results were performed and mean± SEM values of six samples pooled from three experiments are shown. ^*^P < 0.05, compared with the controls; unpaired *t*-tests were used. **(C)** Single-cell suspensions of spleen cells from normal and *S. japonicum* infected mice were stimulated with or without PMA and ionomycin. Expressions of IL-21, IL-10, IL-17, IFN-γ, IL-4, and IL-5 were detected in Tfh cells by FACS analysis. Numbers in quadrants are percentages of cells in each expression phenotype (*n* = 5 mice per group). A representative of two independent experiments is shown. ^*^P < 0.05, compared with the controls.

Next, the isolated splenocytes from both normal and infected mice were stained by different fluorescence labeled antibodies, and the expression of activation-related molecules CD25 and CD69 ICOS were detected on splenic CD4^+^CXCR5^+^PD-1^+^ Tfh cells by using FACS. As shown in Figure 2A, the expression of CD69 [mean fluorescence intensity (MFI) value] on the surface of Tfh cells from the infection group was significantly higher than that of the control group (*P* < 0.05). There was no significant difference in CD25 expression between normal and infected mice (*P* > 0.05). In addition, Tfh cell function associated molecule ICOS was detected on the surface of splenocytes from both normal and infected mice. The results showed that ICOS expression was up-regulated on the Tfh cells of infected mice (*P* < 0.05, Figure 2B).

Moreover, splenocytes from both normal and infected mice were isolated and stimulated by PMA plus ionomycin, and intracellular cytokine staining was done. The ability of Tfh cells to produce cytokines, including IL-21, IL-17, IL-10, IFN-γ, IL-4, and IL-5, was detected by FACS. As shown in Figure 2C, results show that infection induced Tfh cells has significantly improved the ability to produce IL-21, IL-17, and IFN-γ, especially IL-21, compared with control Tfh cells (*P* < 0.05).

### Tfh Cells From *S. japonicum* Infected Mice Promote B Cell Responses

**Figure 3:**
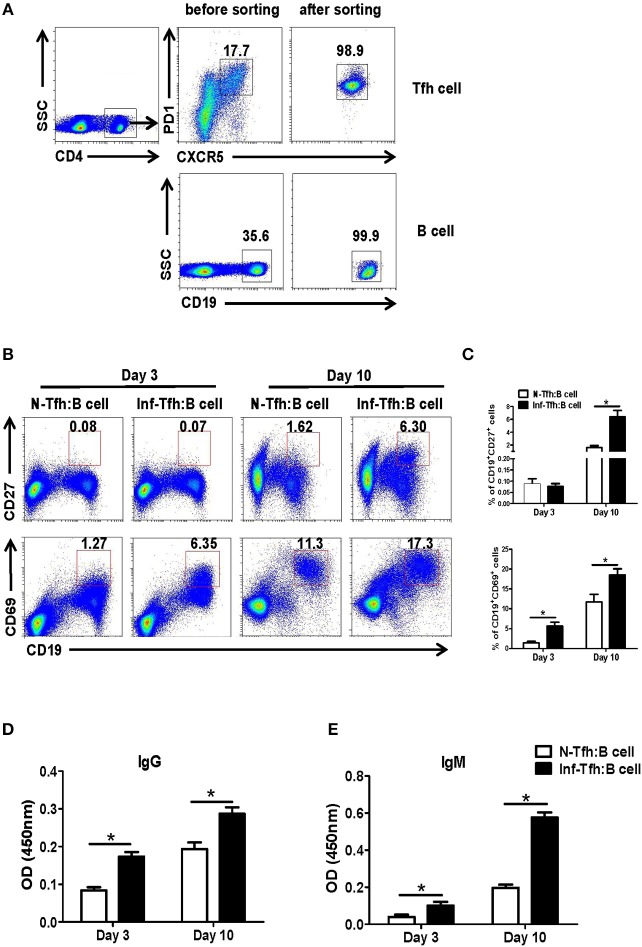
Function of Tfh cells from *S. japonicum* infected mice. **(A)** CD4^+^CXCR5^+^PD-1^+^ Tfh cells from normal or infected mice and allogeneic CD19^+^ B cells from normal controls are incubated at a ratio of 1:1 in RPMI1640 complete medium. **(B,C)** After 3 and or days of culture, the B cell subpopulations are examined based on surface phenotype. **(B)** Representative staining of CD27 and CD69 on B cells from naive mice at days 3 and 10 following co-culture with Tfh cells from normal and *S. japonicum* infected mice. **(D,E)** Mean ± SEM of one representative experiment. IgM and IgG concentration in the supernatant at day 3 or 10. Data are shown as mean ± SEM of 4–6 samples in each group from one representative experiment. ^*^P < 0.05, ^**^P < 0.01 compared with the corresponding controls; unpaired *t*-tests were used.

We further investigated the ability of infection-induced Tfh cells to promote B cell responses. CD4^+^CXCR5^+^PD-1^+^ Tfh cells from spleen of both normal and *S. japonicum* infected mice were sorted out with FACS and co-cultured with B cells isolated from spleen of normal mice, at the ratio of 1:1 in complete medium (Figure 3A). At 3 or 10 days later, the state of maturity and activation of B cells was detected by FACS. Results showed that both CD27 and CD69 expression levels were significantly elevated 10 days later (*P* < 0.05, Figures 3B,C). The supernatant of cultured cells was also collected at days 3 and 10, and the levels of IgG and IgM were detected with ELISA. Compared with normal Tfh cells, infected Tfh cells significantly increased the ability of B cells to secrete IgG and IgM antibodies (*P* < 0.05, Figures 3D,E).

### *S. japonicum* Infection Reduce Apoptosis Related to the Caspase-3 Pathway

**Figure 4:**
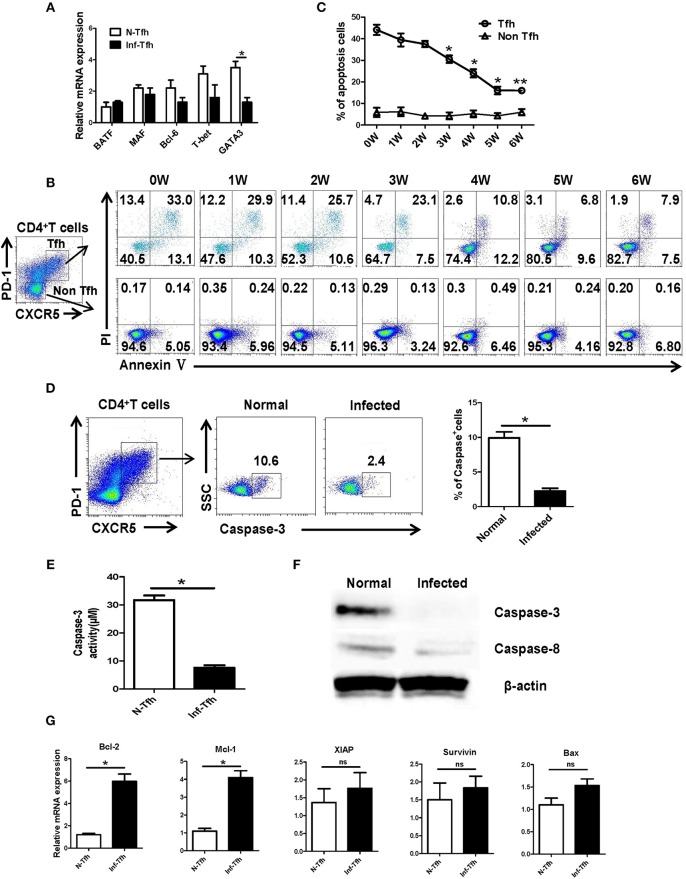
*S. japonicum* infection reduces apoptosis via caspase-3 pathway *in vivo*. **(A)** In spleen Tfh cells purified from normal or *S. japonicum* infected mice, gene expression was determined by qRT-PCR. **(B,C)** Single-cell suspensions of spleens were isolated from in different time points infected mice, and apoptosis was detected using Annexin V-FITC/PI double staining on CD4^+^CXCR5^+^PD-1^+^ Tfh cells and CD4^+^CXCR5^−^PD-1^−^ non-Tfh cells by flow cytometry. **(D)** The percentage of caspase-3^+^ Tfh cells in the spleen of normal and *S. japonicum* infected mice. Representative images (left) and pooled data (right) were shown. **(E)** The caspase-3 activity of the spleen Tfh cells purified from normal or *S. japonicum* infected mice (^*^P < 0.05). **(F)** In spleen Tfh cells purified from normal or *S. japonicum* infected mice, caspase-3 and caspase-8 expression was determined by Western blotting. **(G)** Quantitative RT-PCR analysis of Mcl-1, XIAP, BCL-2, BAX, and Survivin expression in spleen Tfh cells purified from normal or *S. japonicum* infected mice. The data are relative levels of expression compared with those of control cells after normalization with β-actin expression. Data are shown as mean ± SEM of six samples in each group from one representative experiment. ^*^P < 0.05; ns, not significant (*P* > 0.05); unpaired *t*-test.

Next, we investigated how *S. japonicum* infection induce Tfh cells apoptosis. First, CD4^+^CXCR5^+^PD-1^+^ Tfh cells in the spleen of normal and infected mice were separated, and the expressions of important Tfh cell differentiation-related transcription factors were detected with qRT-PCR. No significant difference was detected in the expression of BATF, Bcl-6, and T-bet (*P* > 0.05) except the decreasing of GATA-3 (*P* < 0.05, Figure 4A). We further examined the dynamic apoptosis of Tfh cells after infection. Interestingly, results showed that the apoptosis rate of Tfh cells in the spleen of infected mice was significantly decreased compared with that in normal mice (0W) (*P* < 0.05), while the apoptosis of CD4^+^CXCR5^−^PD-1^−^ non-Tfh cells was not significantly changed (*P* > 0.05, Figures 4B, C). The expression of the key apoptotic protein caspase-3 in Tfh cells in the spleen of both normal and infected mice was further examined with FACS. The expression of caspase-3 in Tfh cells from infected mice was also significantly down-regulated (*P* < 0.05, Figure 4D). Caspase-3 activity was also detected in Tfh cells separated from the spleen of both normal and infected mice by the Caspase-3 Activity Assay Kit as described in materials and methods. Results showed that the activity of caspase-3 was decreased in Tfh cells from infected mice (*P* < 0.05, Figure 4E). In addition, the expressions of caspase-3 and caspase-8 in splenic Tfh cells from both normal and infected mouse were detected by western blotting (WB). Results (Figure 4F) showed that the expression of caspase-3 was significantly decreased in infected Tfh cells (*P* < 0.05), while the expression of caspase-8 only slightly decreased (*P* > 0.05). Next, the expression of apoptosis associated molecules (Mcl-1, XIAP, BcL-2, BAX, Survivin) on the splenic Tfh cells from both normal and infected mice were explored with the use of qRT-PCR. Results (Figure 4G) showed that the expressions of BcL-2 and Mcl-1 (anti-apoptotic proteins) were increased significantly (*P* < 0.05). However, there was no significant difference in the expressions of XIAP, Survivin, and proapoptotic protein BAX (*P* > 0.05).

### SEA Reduces Apoptosis by the Caspase-3 Pathway *in vitro*

**Figure 5:**
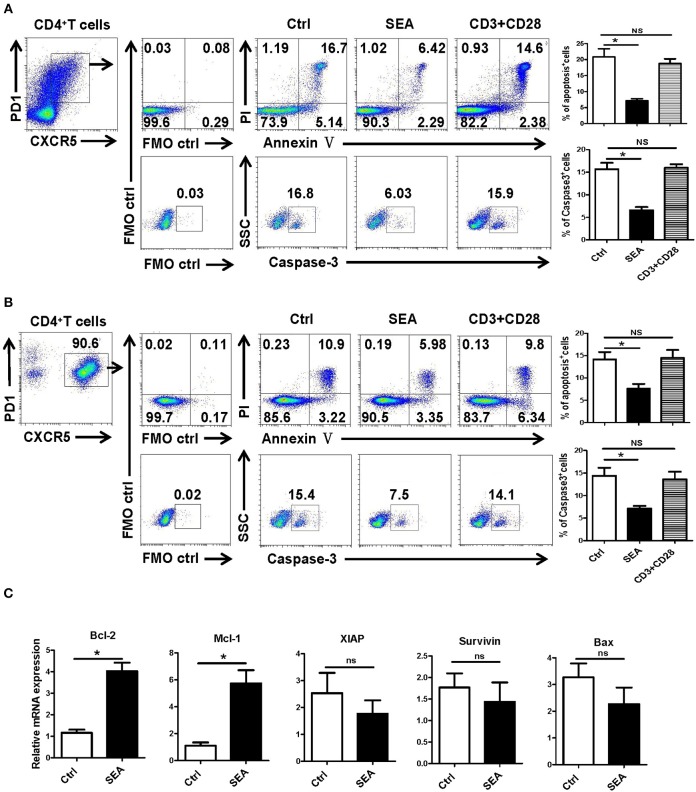
SEA reduces apoptosis via caspase-3 pathway *in vitro*. **(A,B)** Mouse spleen cells **(A)** or Tfh cells **(B)** purified from spleen in normal mice were cultured in medium containing IL-21 (10 ng/ml) with SEA (100 μg/mL) or PBS (control); vehicle was used as control. Apoptosis was detected using Annexin V-FITC/PI double staining with flow cytometry. (Left) Representative results from three independent experiments. (Right) Mean ± SEM values from three independent experiments. **(C)** Tfh cells were cultured in IL21 with the indicated treatments for 24 h; mRNA expression of Mcl-1, XIAP, BCL-2, BAX, and Survivin was determined by qRT-PCR. The data are relative levels of expression compared with those of control cells after normalization with β-actin expression. Data are shown as mean ± SEM of three to five samples in each group from one representative experiment. ^*^P < 0.05; ns, not significant (*P* > 0.05); unpaired *t*-test.

We further explored the causes of Tfh aggregation after *S. japonicum* infection *in vitro*. SEA was added to stimulate the splenocytes isolated from normal mice, and the non-stimulated control was set. After 24 h of treatment, the apoptosis and caspase-3 expressions of Tfh were analyzed by flow cytometry. The results showed that SEA can significantly inhibit the apoptosis of Tfh and the expression of caspase-3 (*P* < 0.05, Figure 5A). In order to further verify these results, Tfh cells in the spleen of normal mice were separated and treated with SEA *in vitro*. Apoptosis and caspase-3 expressions of Tfh were analyzed by flow cytometry 24 h later. Consistent with these results, SEA could significantly inhibit the apoptosis of Tfh and the expression of caspase-3 (*P* < 0.05, Figure 5B). At the same time the stimulated cells were collected, mRNA was extracted. The expression of apoptosis-related genes was detected with qRT-PCR. Results (Figure 5C) show a significant increase in anti-apoptotic factor BcL-2 and Mcl-1 (*P* < 0.05) and no obvious difference in the expression of anti-apoptotic genes *XIAP* and *survivin* and apoptosis-promoting factor BAX in SEA-stimulated cells (*P* > 0.05).

### Effects of SEA on Tfh Cells Are Dependent on the Caspase-3 Pathway

**Figure 6:**
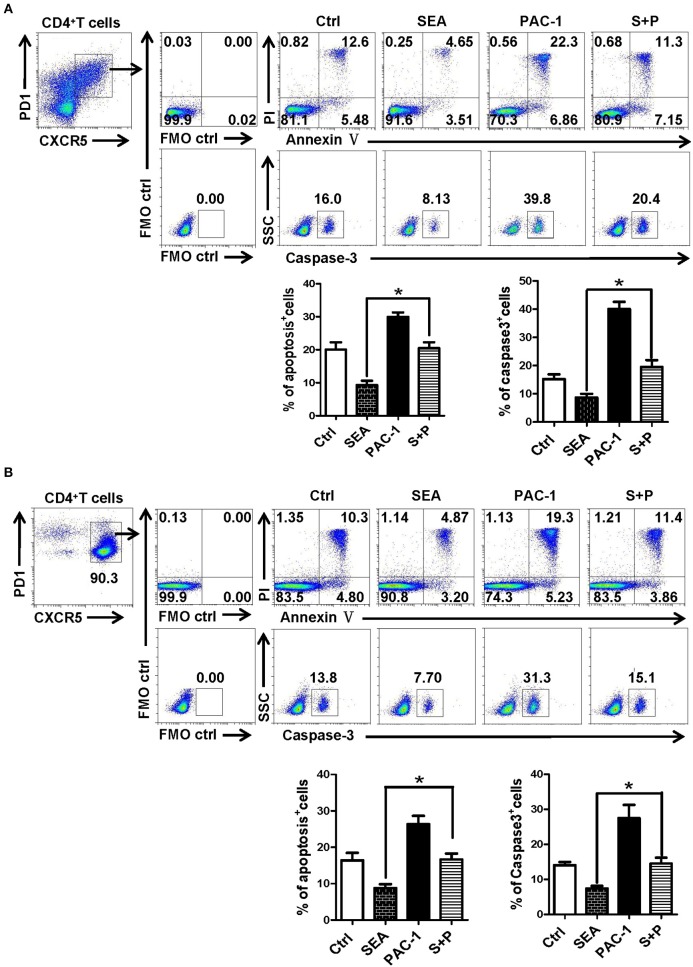
SEA reduces apoptosis via caspase-3 pathway *in vitro*. **(A,B)** Splenocytes **(A)** was isolated from normal mice, and CD4^+^CXCR5^+^PD-1^+^ Tfh cells was purified by FACS. The splenocytes and Tfh cells were cultured in medium containing IL-21 (10 ng/ml) with SEA (100 μg/mL), PAC-1 (100 μM/mL); vehicle was used as control. Apoptosis was detected using Annexin V-FITC/PI double staining with flow cytometry. (Left) Representative results from three to five independent experiments. (Right) Data are shown as mean ± SEM of six samples in each group from one representative experiment, and repeat three times with similar results. ^*^P < 0.05.

In order to further explore the action of SEA on the apoptosis of Tfh cells, Tfh cells and lymphocytes from the spleen of normal mice were stimulated by SEA (SEA group), caspase-3 agonist PAC-1 (PAC-1 group), and SEA plus PAC-1 (S+P group); solvent control was also set. At 12 h later, the apoptosis and expression of caspase-3 of Tfh cells were detected by FACS. Results showed that apoptosis and caspase-3 expressions of Tfh cells in the SEA group were significantly lower than those in the control group (*P* < 0.05, Figure 6). As an agonist of caspase-3, the PAC-1 could significantly up-regulated the expression of caspase-3 and cell apoptosis (*P* < 0.05). However, the addition of SEA could directly inhibit the up-regulation of caspase-3 expression and the apoptosis of Tfh cells induced by PAC-1 (*P* < 0.05).

## Discussion

Follicular helper T cell is an effector CD4^+^ T cell subgroup that plays an important role in both protection and pathological immune response. Our experimental results demonstrated that after *S. japonicum* infection, the proportion and absolute number of CD4^+^CXCR5^+^PD-1^+^ Tfh cells in the spleen, lymph nodes, and peripheral blood of mice increased significantly (*P* < 0.01), especially in the spleen. The percentage of splenic Tfh cell increased sharply from week 3 to 5 after infection (Figure 1). These results indicated that *S. japonicum* infection could induce the aggregation of Tfh cells, and spleen might be the main deposition organs for these infections induced Tfh cells. This results consistented with Chen et al. have reported (13). It implied that Tfh cells might involve in *S. japonicum* infection induced tissue damages.

The spleen is the largest peripheral immune organ in the body and the main organ for B cell somatic cell mutation, affinity maturation, receptor editing and type transformation, and other processes through which immune effects are finally exerted (30). The phonotypic and functional changes of the Tfh cells were further explored in the spleen of *S. japonicum* infected mice. As shown in Figure 2, compared with normal Tfh cells, the expression of CD69 and ICOS were significantly up-regulated in Tfh cells from infected mice (*P* < 0.05, Figures 2A,B). It demonstrated that *S. japonicum* infection could induce the activation of Tfh cells in the spleen of mice. Tfh cells can secrete a variety of cytokines to play a regulatory role after activation (31, 32). IL-21 was the main cytokine produced by Tfh cells after activation and is the key cytokine for Tfh cells to assist B cells (33). Our results (Figure 2C) showed that *S. japonicum* infection significantly increased the ability of Tfh cells to secrete IL-21, IL-17, and IFN- γ (*P* < 0.05). It further suggested that Tfh cell is a multi-functional cell, which could play a variety of important roles in the course of *S. japonicum* infection.

Tfh cells have showed important roles in promoting B lymphocytes proliferation and differentiation, helping antibody production and classification transformation (1, 34). To investigate the ability of infection-induced Tfh cells to promote B cell responses, Tfh cells from the spleen of both normal and *S. japonicum* infected mice were sorted out by FACS and co-cultured with B cells isolated from spleen of normal mice. As shown in Figure 3, our results indicated that Tfh cells separated from infected mice could assist in B cell activation more quickly and strongly (*P* < 0.05), especially the differentiation of the CD19^+^CD27^+^ memory B cells (*P* < 0.05). Moreover, it could increase the content of IgG and IgM in the supernatant (*P* < 0.05). These results suggested that *S. japonicum* infection induced Tfh cells could directly promote B cell activation and differentiation *in vitro*. It means that splenic Tfh cells play an important role in the course of *S. japonicum* infection induced immune response.

BATF and MAC, T-bet, GATA-3 are lymphocytes activation and differentiation related molecules (35–37). As shown in Figure 4A, only the expression of *GATA-3*, the classic Th2-related gene (37), was decreased (*P* < 0.05). It was reported that infection of *S. mannii* could promote the apoptosis of CD4^+^ T cells (27). As shown in Figures 4B,C, our dynamic results indicated that continual decreasing of apoptosis was found in splenic Tfh cells from *S. japonicum* infected mice (from week 3 to 6, *P* < 0.05). It suggests that the accumulation of Tfh cells after infection is closely related to the down-regulating apoptosis of Th cells.

Caspase-3 is downstream of the apoptotic enzyme cascade reaction, which directly performs the task of causing cell death (38). This protein is involved in the hydrolysis of cellular proteins required for apoptosis, which lead to the lysis of functional proteins and mediate the occurrence of apoptosis (39). Both qRT-PCR and Western results (Figures 4D–F) demonstrate that the expression of caspase-3 in Tfh cells significantly decreased after *S. japonicum* infection (*P* < 0.05). It implied that the decrease in apoptosis of Tfh cell after infection might regulated by caspase-3. Moreover, the expression of caspase-3-regulated apoptosis-related genes (BcL-2, Mcl-1, XIAP, Survivin, and BAX) was compared between Tfh cells from normal and infected mice. Results showed that the expression of anti-apoptosis factors Mcl-1 and Bcl-2 on Tfh cells from infected mice was obviously higher (Figure 4F, *P* < 0.05). It suggested that the decrease in apoptosis of Tfh cells might induced by the up-regulation of anti-apoptosis factors Mcl-1 and BcL-2 in this model.

SEA is the initial factor in the formation of granuloma, and it has many regulatory effects on the immune response of the host (40, 41). It was reported that SEA immunization could induce obvious IL-4 and IL-21 secreting Tfh cells in the reactive LN, which could enhance GC development and class switching (42). Studies have shown that SEA can induce the apoptosis of activated HSCs through the Akt/P53/DR5 signaling pathway and caspase-3/8 signaling pathway (43). Thus, SEA was used to stimulate splenoctes and the purified splenic Tfh cells *in vitro*. As shown in Figures 5A,B, our results indicate that Tfh cell apoptosis and caspase-3 expression were significantly decreased after 12 h stimulation by SEA (*P* < 0.05). It indicates that SEA could directly suppress the apoptosis of Tfh cells mediated by caspase-3. The expression of apoptosis and anti-apoptosis-related molecules induced by SEA was consistent with the *in vivo* results (Figure 5C).

PAC-1 is the first discovered regulatory gene that can be directly applied to procaspase-3 small molecule compounds, can activate cell suicide process, cause apoptosis (44). To further evaluate the effect of SEA on caspase-3 expression and Tfh cells apoptosis, SEA was used to simulate PAC-1 cultured splenocytes and purified Tfh cells. As shown in Figures 6A,B, SEA could directly inhabit PAC-1 induced Tfh cell apoptosis and caspase-3 expression significantly (*P* < 0.05). It confirms that SEA promotes the aggregation of Tfh cells by inhibiting the expression of caspase-3 and down-regulating the apoptosis of Tfh cells.

In conclusion, this study indicated that SEA could induce the aggregation of Tfh cells in the spleen of *S. japonicum* infected C57BL/6 mouse, which then activate and interact with B cells. Inhibiting the apoptosis of Tfh cells by down-regulating the expression of caspase-3 is the main mechanism for Tfh cell aggregation.

## Ethics Statement

This study was carried out in accordance with the recommendations of Administration of Affairs Concerning Experimental Animals Institutional Animal Committee of Guangzhou Medicine University, name of committee. The protocol was approved by the institutional animal care and use committee of Guangzhou Medical University.

## Author Contributions

QY and JQ performed most experiments and analyzed data with the support from JH. CJ, YF, and HX performed animal experiment. JZ, GL, and SX performed parasite infection experiment. HQ and YQ contributed to scientific planning. QY and JH oversaw and designed the study. QY, JH, and JM wrote the manuscript.

### Conflict of Interest Statement

The authors declare that the research was conducted in the absence of any commercial or financial relationships that could be construed as a potential conflict of interest.

## References

[1] Lo NC Gurarie D Yoon N Coulibaly JT Bendavid E Andrews JR. Impact and cost-effectiveness of snail control to achieve disease control targets for schistosomiasis. Proc Natl Acad Sci USA. (2018) 115:E584–91. 10.1073/pnas.1708729114 29301964 PMC5789907

[2] Ricciardi A Dalton JP Ndao M. Evaluation of the immune response and protective efficacy of *Schistosoma mansoni* Cathepsin B in mice using CpG dinucleotides as adjuvant. Vaccine. (2015) 33:346–53. 10.1016/j.vaccine.2014.11.016 25448114

[3] Zhang Y Zhang J Bo SY Wang GZ Xin XF. Observation on dynamic changes of SEA specific antibody in sera of BALB/c mice infected with *Schistosoma japonicum*. Zhongguo Xue Xi Chong Bing Fang Zhi Za Zhi. (2012) 24:284–89. 23012950

[4] Chen D Luo X Xie H Gao Z Fang H Huang J. Characteristics of IL-17 induction by *Schistosoma japonicum* infection in C57BL/6 mouse liver. Immunology. (2013) 139:523–32. 10.1111/imm.12105 23551262 PMC3719069

[5] Chen D Xie H Cha H Qu J Wang M Li L. Characteristics of *Schistosoma japonicum* infection induced IFN-gamma and IL-4 co-expressing plasticity Th cells. Immunology. (2016) 149:25–34. 10.1111/imm.12623 27242265 PMC4981615

[6] Li L Xie H Wang M Qu J Cha H Yang Q. Characteristics of IL-9 induced by *Schistosoma japonicum* infection in C57BL/6 mouse liver. Sci Rep. (2017) 7:2343. 10.1038/s41598-017-02422-8 28539607 PMC5443805

[7] Yang Q Qiu H Xie H Qi Y Cha H Qu J. A *Schistosoma japonicum* infection promotes the expansion of myeloid-derived suppressor cells by activating the JAK/STAT3 pathway. J Immunol. (2017) 198:4716–27. 10.4049/jimmunol.1601860 28476935

[8] Crotty S. T follicular helper cell differentiation, function, and roles in disease. Immunity. (2014) 41:529–42. 10.1016/j.immuni.2014.10.004 25367570 PMC4223692

[9] Hong JJ Chang KT Villinger F. The dynamics of T and B cells in lymph node during chronic HIV infection: TFH and HIV, unhappy dance partners? Front Immunol. (2016) 7:522. 10.3389/fimmu.2016.00522 27920778 PMC5118424

[10] Papp G Szabo K Szekanecz Z Zeher M. Follicular helper T cells in autoimmune diseases. Rheumatology. (2014) 53:1159–60. 10.1093/rheumatology/ket434 24402581

[11] Kong FY Feng B Zhang HH Rao HY Wang JH Cong X. CD4+CXCR5+ T cells activate CD27+IgG+ B cells via IL-21 in patients with hepatitis C virus infection. Hepatobiliary Pancreat Dis Int. (2016) 15:55–64. 10.1016/S1499-3872(16)60054-1 26818544

[12] Huang S Cao Y Lu M Peng W Lin J Tang C. Identification and functional characterization of *Oncomelania hupensis* macrophage migration inhibitory factor involved in the snail host innate immune response to the parasite *Schistosoma japonicum*. Int J Parasitol. (2017) 47:485–99. 10.1016/j.ijpara.2017.01.005 28322847

[13] Chen X Yang X Li Y Zhu J Zhou S Xu Z. Follicular helper T cells promote liver pathology in mice during *Schistosoma japonicum* infection. PLoS Pathog. (2014) 10:e1004097. 10.1371/journal.ppat.1004097 24788758 PMC4006917

[14] Sharma R Das A IL-2 mediates NK cell proliferation but not hyperactivity. Immunol Res. (2018) 66:151–7. 10.1007/s12026-017-8982-3 29256180

[15] Xu K Liu XF Ke ZQ Yao Q Guo S Liu C. Resveratrol modulates apoptosis and autophagy induced by high glucose and palmitate in cardiac cells. Cell Physiol Biochem. (2018) 46:2031–40. 10.1159/000489442 29723857

[16] Shao B Fu X Yu Y Yang D. Regulatory effects of miRNA181a on FasL expression in bone marrow mesenchymal stem cells and its effect on CD4+T lymphocyte apoptosis in estrogen deficiencyinduced osteoporosis. Mol Med Rep. (2018) 18:920–30. 10.3892/mmr.2018.9026 29845202 PMC6059724

[17] Huang H An Y Jiao W Wang J Li S Teng X. CHOP/caspase-3 signal pathway involves in mitigative effect of selenium on lead-induced apoptosis via endoplasmic reticulum pathway in chicken testes. Environ Sci Pollut Res Int. (2018) 25:18838–45. 10.1007/s11356-018-1950-1 29713980

[18] O'Donovan N Crown J Stunell H Hill AD McDermott E O'Higgins N. Caspase 3 in breast cancer. Clin Cancer Res. (2003) 9:738–42. 12576443

[19] Ashokkumar C Sun Q Ningappa M Higgs BW Mazariegos G Zeevi A. Antithymocyte globulin facilitates alloreactive T-cell apoptosis by means of caspase-3: potential implications for monitoring rejection-free outcomes. Transplantation. (2015) 99:164–70. 10.1097/TP.0000000000000289 25531894 PMC4274752

[20] Wang F Wang L Li Y Wang N Wang Y Cao Q. PAC-1 and its derivative WF-210 Inhibit Angiogenesis by inhibiting VEGF/VEGFR pathway. Eur J Pharmacol. (2018) 821:29–38. 10.1016/j.ejphar.2017.12.035 29269017

[21] Shi X Chen X Li X Lan X Zhao C Liu S. Gambogic acid induces apoptosis in imatinib-resistant chronic myeloid leukemia cells via inducing proteasome inhibition and caspase-dependent Bcr-Abl downregulation. Clin Cancer Res. (2014) 20:151–63. 10.1158/1078-0432.CCR-13-1063 24334603 PMC3938960

[22] Rahmani M Aust MM Attkisson E Williams DJ Ferreira-Gonzalez A Grant S. Dual inhibition of Bcl-2 and Bcl-xL strikingly enhances PI3K inhibition-induced apoptosis in human myeloid leukemia cells through a GSK3- and Bim-dependent mechanism. Cancer Res. (2013) 73:1340–51. 10.1158/0008-5472.CAN-12-1365 23243017 PMC3578060

[23] Ke F Vanyai HK Cowan AD Delbridge A Whitehead L Grabow S. Embryogenesis and adult life in the absence of intrinsic apoptosis effectors BAX, BAK, and BOK. Cell. (2018) 173:1217–30. 10.1016/j.cell.2018.04.036 29775594

[24] Khanzadeh T Hagh MF Talebi M Yousefi B Azimi A Hossein PFA. Investigation of BAX and BCL2 expression and apoptosis in a resveratrol- and prednisolone-treated human T-ALL cell line, CCRF-CEM. Blood Res. (2018) 53:53–60. 10.5045/br.2018.53.1.53 29662863 PMC5898995

[25] Chen C Liu TS Zhao SC Yang WZ Chen ZP Yan Y. XIAP impairs mitochondrial function during apoptosis by regulating the Bcl-2 family in renal cell carcinoma. Exp Ther Med. (2018) 15:4587–93. 10.3892/etm.2018.5974 29731840 PMC5920643

[26] Woo SM Seo SU Min KJ Kwon TK. BIX-01294 sensitizes renal cancer Caki cells to TRAIL-induced apoptosis through downregulation of survivin expression and upregulation of DR5 expression. Cell Death Discov. (2018) 4:29. 10.1038/s41420-018-0035-8 29531826 PMC5841352

[27] Lundy SK Lerman SP Boros DL. Soluble egg antigen-stimulated T helper lymphocyte apoptosis and evidence for cell death mediated by FasL(+) T and B cells during murine *Schistosoma mansoni* infection. Infect Immun. (2001) 69:271–80. 10.1128/IAI.69.1.271-280.2001 11119515 PMC97881

[28] Wang J Xu F Zhu D Duan Y Chen J Sun X. *Schistosoma japonicum* soluble egg antigens facilitate hepatic stellate cell apoptosis by downregulating Akt expression and upregulating p53 and DR5 expression. PLoS Negl Trop Dis. (2014) 8:e3106. 10.1371/journal.pntd.0003106 25144704 PMC4140669

[29] Duan Y Gu X Zhu D Sun W Chen J Feng J. *Schistosoma japonicum* soluble egg antigens induce apoptosis and inhibit activation of hepatic stellate cells: a possible molecular mechanism. Int J Parasitol. (2014) 44:217–24. 10.1016/j.ijpara.2013.11.003 24487000

[30] Sato K Honda SI Shibuya A Shibuya K. Cutting edge: identification of marginal reticular cells as phagocytes of apoptotic B cells in germinal centers. J Immunol. (2018) 200:3691–6. 10.4049/jimmunol.1701293 29686051

[31] Leavy O. T cells: the TFH-like transition of TH1 cells. Nat Rev Immunol. (2012) 12:74. 10.1038/nri3161 22273767

[32] Ma CS Deenick EK Batten M Tangye SG. The origins, function, and regulation of T follicular helper cells. J Exp Med. (2012) 209:1241–53. 10.1084/jem.20120994 22753927 PMC3405510

[33] Xu S Xue X You K Fu J. Caveolin-1 regulates the expression of tight junction proteins during hyperoxia-induced pulmonary epithelial barrier breakdown. Respir Res. (2016) 17:50. 10.1186/s12931-016-0364-1 27176222 PMC4866358

[34] Schulzke JD Ploeger S Amasheh M Fromm A Zeissig S Troeger H. Epithelial tight junctions in intestinal inflammation. Ann N Y Acad Sci. (2009) 1165:294–300. 10.1111/j.1749-6632.2009.04062.x 19538319

[35] Liao J Humphrey SE Poston S Taparowsky EJ. Batf promotes growth arrest and terminal differentiation of mouse myeloid leukemia cells. Mol Cancer Res. (2011) 9:350–63. 10.1158/1541-7786.MCR-10-0375 21296860 PMC3060294

[36] Swoboda S Gruettner J Lang S Wendel HP Beyer ME Griesel E. Expression of CD11b (MAC-1) and CD162 (PSGL-1) on monocytes is decreased under conditions of deep hypothermic circulatory arrest. Exp Ther Med. (2014) 8:488–92. 10.3892/etm.2014.1737 25009606 PMC4079448

[37] Das A Ranganathan V Umar D Thukral S George A Rath S. Effector/memory CD4 T cells making either Th1 or Th2 cytokines commonly co-express T-bet and GATA-3. PLoS ONE. (2017) 12:e185932. 10.1371/journal.pone.0185932 29088218 PMC5663332

[38] LeBlanc AC. Natural cellular inhibitors of caspases. Prog Neuropsychopharmacol Biol Psychiatry. (2003) 27:215–29. 10.1016/S0278-5846(03)00017-4 12657361

[39] Creagh EM Conroy H Martin SJ. Caspase-activation pathways in apoptosis and immunity. Immunol Rev. (2003) 193:10–21. 10.1034/j.1600-065X.2003.00048.x 12752666

[40] Peng H Zhang Q Li X Liu Z Shen J Sun R. IL-33 contributes to *Schistosoma japonicum*-induced hepatic pathology through induction of M2 macrophages. Sci Rep. (2016) 6:29844. 10.1038/srep29844 27445267 PMC4956744

[41] Hirata M Kage M Hara T Nakao M Fukuma T. Inhibitory effect of circulating egg antigens on *Schistosoma japonicum* egg-induced granuloma formation. J Parasitol. (1997) 83:842–7. 10.2307/3284278 9379288

[42] Fairfax KC Everts B Amiel E Smith AM Schramm G Haas H IL-4-secreting secondary T follicular helper (Tfh) cells arise from memory T cells, not persisting Tfh cells, through a B cell-dependent mechanism. J Immunol. (2015) 194:2999–3010. 10.4049/jimmunol.1401225 25712216 PMC4495582

[43] Gaber HM Maghraby AS Ahmed MB Ruppel A Bahgat MM. Immune responses in mice after immunization with antigens from different stages of the parasite *Schistosoma mansoni*. Z Naturforsch C. (2010) 65:289–302. 10.1515/znc-2010-3-419 20469651

[44] Putt KS Chen GW Pearson JM Sandhorst JS Hoagland MS Kwon JT. Small-molecule activation of procaspase-3 to caspase-3 as a personalized anticancer strategy. Nat Chem Biol. (2006) 2:543–50. 10.1038/nchembio814 16936720

